# In Vitro and in Vivo antitumor activity and the mechanism of siphonodictyal B in human colon cancer cells

**DOI:** 10.1002/cam4.2409

**Published:** 2019-07-31

**Authors:** Sonoko Chikamatsu, Ken Saijo, Hiroo Imai, Koichi Narita, Yoshifumi Kawamura, Tadashi Katoh, Chikashi Ishioka

**Affiliations:** ^1^ Department of Clinical Oncology, Institute of Development, Aging and Cancer Tohoku University Sendai Japan; ^2^ Department of Medical Oncology Tohoku University Hospital Sendai Japan; ^3^ Laboratory of Synthetic and Medicinal Chemistry, Faculty of Pharmaceutical Sciences Tohoku Medical and Pharmaceutical University Sendai Japan; ^4^ Faculty of Pharmaceutical Sciences, Research Center for Drug Discovery Tohoku Medical and Pharmaceutical University Sendai Japan

**Keywords:** liphagal, p38 MAPK pathway, reactive oxygen species, siphonodictyal B, terpenoid

## Abstract

Liphagal, isolated from the marine sponge *Aka coralliphaga*, exhibits phosphatidylinositol 3‐kinase alpha (PI3K*α*) inhibitory activity and cytotoxic effects in human cancer cells. Siphonodictyal B, the biogenetic precursor of liphagal, also has PI3K inhibitory activity. However, its cytotoxic or antitumor activities have not been evaluated. In this study, we demonstrated that siphonodictyal B inhibits several kinases such as CDK4/6, CDK7, and PIM2 in addition to PI3K in vitro and that siphonodictyal B exhibits more potent cytotoxic effects than liphagal against human colon cancer cell lines. Furthermore, treatment with siphonodictyal B resulted in increased PARP cleavage, a larger sub‐G1 fraction, and a larger annexin V‐positive cell population, all of which are indicative of apoptosis induction. As a mechanism of apoptosis induction, we found that siphonodictyal B activates the p38 MAPK pathway, leading the upregulation of proapoptotic factors. Moreover, siphonodictyal B increased ROS levels, thus promoting p38 MAPK pathway activation. NAC, an ROS scavenger, almost completely reversed both the cytotoxic and p38 MAPK pathway‐activating effects of siphonodictyal B. These results indicate that the p38 MAPK pathway might be involved downstream of ROS signaling as part of the mechanism of siphonodictyal B‐induced apoptosis. Finally, siphonodictyal B displayed antitumor effects in a human colon cancer xenograft mouse model and increased p38 phosphorylation in tumor tissue. These results suggest that siphonodictyal B could serve as the basis of a novel anticancer drug.

## Graphical abstract

Siphonodictyal B induces apoptosis via activation of p38 MAPK pathway in human colorectal cancer cells.

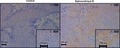

## 1 INTRODUCTION

**Figure 1:**
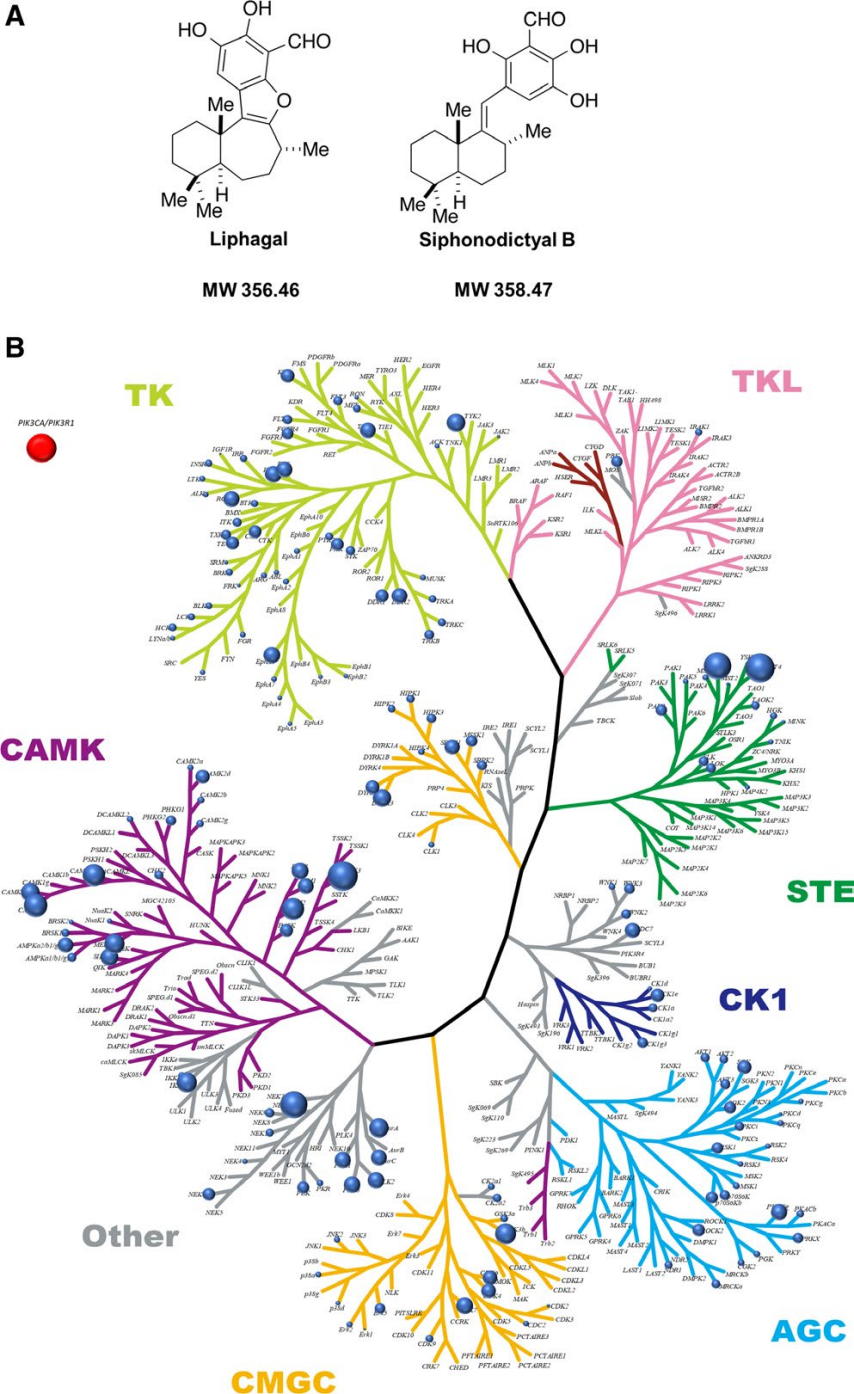
Chemical structure and kinome inhibition plot. A, Chemical structure of liphagal and siphonodictyal B. B, Plot of kinome inhibition by siphonodictyal B. The inhibition of 313 protein kinases by siphonodictyal B at 10 μmol/L was evaluated using the off‐chip mobility shift assay or immobilized metal ion affinity‐based fluorescence polarization assay. The strengths of the inhibitory activities against kinases are illustrated by the sizes of the circles. PI3K was not included in the tree because of its lipid kinase features. Therefore, the circle was written in a separate frame

Compounds isolated from natural products have attracted attention, as some possess unique chemical structures and biological activities that can lead to the development of novel anticancer drugs.^1^, ^2^, ^3^ Terpenoids are the most widespread group of natural products with a chemically characteristic structure of five‐carbon isoprene units.^1^, ^2^ Liphagal and siphonodictyal B are meroterpenoids that were isolated from the marine sponge *Aka coralliphaga* 4, 5 (Figure 1). Generally, it is difficult to obtain sufficient samples of natural products for biological assays or developing pharmaceutical products because of their scarcity. Meanwhile, the total synthesis of such natural compounds has been pursued with great enthusiasm. Concerning liphagal and siphonodictyal B, efficient and successful total synthetic methods have been reported.^5^ In the synthetic process, siphonodictyal B corresponds to the biogenetic precursor of liphagal.^5^

Furthermore, both liphagal and siphonodictyal B have been reported to exert inhibitory activity against phosphatidylinositol 3‐kinase (PI3K),^4^, ^6^ a lipid kinase that converts phosphatidylinositol 4,5‐bisphosphate to phosphatidylinositol 3,4,5‐triphosphate (PIP3). PIP3 in turn activates protein kinase B (AKT) and downstream molecules, leading to the promotion of cell growth, proliferation, and survival.^7^ In many types of cancer cells, the PI3K‐AKT pathway is frequently activated through gain‐of‐function mutations in PIK3CA, which encodes p110*α*, the catalytic subunit of PI3K.^8^ Therefore, PI3K is considered a potential drug target for cancer therapy.^9^, ^10^ Although liphagal was reported to exhibit cytotoxic effects in human colon cancer cells,^4^ the effects of siphonodictyal B on human cancer cell have never been evaluated. In the present study, we illustrated that siphonodictyal B induced apoptosis more potently than liphagal in human colon cancer cells. Then, we focused on siphonodictyal B and analyzed the mechanism by which siphonodictyal B induces apoptosis. Furthermore, we confirmed the in vivo antitumor activity of siphonodictyal B in a human colon cancer cell xenograft mouse model.

## 2 MATERIALS AND METHODS

### 2.1 Reagents

Liphagal and siphonodictyal B were synthesized chemically and provided by Tadashi Katoh at Tohoku Medical and Pharmaceutical University (Sendai, Japan). GDC0941 was purchased from Abcam (Cambridge, UK). Palbociclib was purchased from ChemoScene (Monmouth Junction, NJ, USA). SB203580 was purchased from Cayman Chemical Company (Ann Arbor, MI, USA). N‐acetyl‐l‐cysteine (NAC) was purchased from Sigma‐Aldrich (St. Louis, MO, USA).

### 2.2 Cell lines and cell culture

The human colon cancer cell lines HCT 116 and SW480 were purchased from American Type Culture Collection (ATCC, Manassas, VA, USA). HT‐29 was a gift from Dr John M. Mariandason at Ludwig Institute for Cancer Research, Austin Hospital (Heidelberg, VIC, Australia). Early passage aliquots were frozen. Cells used in this study were replenished from the frozen stocks. All cell lines were cultured in RPMI‐1640 medium supplemented 10% fetal bovine serum at 37°C in an atmosphere of 5%CO_2_.

### 2.3 Enzyme assay in cell‐free system

Cellular kinase activities were evaluated using nonradioisotopic methods such as the off‐chip mobility shift assay or IMAP (Carna Biosciences, Kobe, Japan).^11^ A kinase inhibition profiling panel was produced on the basis of the kinase inhibition rates. In off‐chip mobility shift assay, compound solution was prepared in assay buffer (20 mmol/L HEPES, 0.01% Triton X‐100, 2 mmol/L DTT, pH 7.5) and incubated in a 384‐well plate at room temperature. The reaction was stopped by adding Termination Buffer (QuickScout Screening Assist MSA; Carna Biosciences, Kobe, Japan). The substrate peptide and the phosphorylated peptide in the reaction solution were separated and quantified by LabChip system (Perkin Elmer, MA, USA). In IMAP assay, compound solution was prepared in assay buffer (20 mmol/L HEPES, 0.01% Tween‐20, 2 mmol/L DTT, pH 7.4) and incubated in a 384‐well black plate at room temperature. IMAP binding reagent (IMAP Screening Express kit; Molecular Devices, CA, USA) was added and incubated for 30 minutes. The kinase reaction was evaluated at the fluorescence polarization.

### 2.4 Cell proliferation assay

Cells were seeded in 96‐well plates at 4000 cells/well and incubated for 24 hours, then treated with each concentration of siphonodictyal B. In experiments with SB203580 or NAC, cells were further preincubated for 24 or 1 hour before application of siphonodictyal B, respectively. Cells were incubated with Cell Counting Kit‐8 (Dojindo Molecular Technologies, Kumamoto, Japan) for 1.5 hours. Then, the absorbance of each well was measured using SpectraMaxM2e (Molecular Devices, CA, USA). The ratios of surviving cells to control cells treated with 0.1% DMSO were calculated.

### 2.5 Cell cycle analysis

A total of 1 × 10^6^ HCT 116 cells were seeded and incubated for 24 hours. Cells were treated with each concentration of siphonodictyal B for 24 hours. Cells were harvested, washed in PBS, and fixed with 70% ethanol overnight at 4°C. After that, cells were incubated with RNase and propidium iodide (PI). The measurement was performed using FC‐500 (Beckman Coulter, CA, USA).

### 2.6 Western blot analysis

HCT 116 cells were seeded in six‐well plates at 2 × 10^5^ cells/well and incubated for 24 houra. Cells were treated with each concentration of siphonidictyal B, GDC0941, palbociclib, or a combination of GDC0941 and palbociclib for 48 hours. Cells were harvested and lysed using RIPA buffer. The proteins were separated on an SDS‐PAGE and transferred to PVDF membranes. Then, the membranes were blocked for 1 hour and incubated with primary antibodies overnight at 4°C. The membranes were washed and incubated with secondary antibodies for 1 hour at room temperature. Next, the resultant bands were detected using an Odyssey Infrared Imaging system (LI‐COR, NB, USA). Antibodies against phospho‐RB (Ser780), AKT, phospho‐AKT (Ser437), PARP, phospho‐p38 MAPK (Thr180/Tyr182), p38 MAPK, and Bim were purchased from Cell Signaling Technology (Danvers, MA, USA). Antibodies against *β*‐actin and *α*‐tubulin were purchased from Sigma‐Aldrich.

### 2.7 siRNA transfection

HCT 116 cells were seeded in 24‐well plates at 2.5 × 10^4^ cells/well and incubated for 24 hours. Cells were transfected with 6 pmol/well siRNA against p38 or negative control siRNA using Lipofectamine 2000 Transfection Reagent (Thermo Fisher Scientific, Waltham, MA, USA). All siRNAs were purchased from Thermo Fisher Scientific.

### 2.8 Annexin V‐PI double staining

HCT 116 cells were seeded in six‐well plates at 2 × 10^5^ cells/well and incubated for 24 hours. In experiments with SB203580 or NAC, cells were further preincubated for 24 or 1 hour before application of siphonodictyal B, respectively. Cells were washed with PBS and stained using an Annexin V‐FITC Apoptosis Detection Kit (Nacalai Tesque, Kyoto, Japan). The measurement was performed using FC‐500.

### 2.9 Reactive oxygen species (ROS) detection

HCT 116 cells were treated with each drug for 2 hours and stained using a DCFDA/H2DCFDA—Cellular Reactive Oxygen Species Detection Assay Kit (Abcam). The measurement was performed using FC‐500.

### 2.10 Xenograft mouse model

All animal experiments were performed according to the Regulations for Animal Experiments and Related Activities at Tohoku University. Six‐week‐old female Balb/c nude mice were purchased from Charles River Laboratories (Wilmington, MA, USA). HCT 116 cells (5 × 10^6^) were implanted subcutaneously into the right flanks of nude mice. After tumor formation was confirmed, mice were assigned to treatment with 20 mg/kg siphonodictyal B or vehicle (four mice per group) administered intraperitoneally every 3 days. Siphonodictyal B was dissolved in saline with 2.5% CremophorEL^®^ and 2.5% ethanol. Tumor volumes were calculated on the basis of tumor mass using the formula length × width × width/2 (mm^3^). The body weights of mice were also measured every 3 days.

### 2.11 Immunohistochemistry

Tumor tissues were collected from mice, fixed in 10% formalin, and embedded in paraffin. Tumor sections on slides were dewaxed, rehydrated, and incubated with a specific antibody against phospho‐p38.

### 2.12 Statistical analysis

All data were presented as the mean ± standard deviation. Statistical analysis was performed using JMP Pro 12 software (SAS, Cary, NC, USA). Unless otherwise noted, the significance of differences was examined using Student's *t* tests. *P* < 0.05 indicated a statistically significant difference.

## 3 RESULTS

### 3.1 Evaluation of the kinase inhibitory activities of liphagal and siphonodictyal B in cell‐free system

Although liphagal and siphonodictyal B were reported to have PI3K (p110*α*/p85*α*) inhibitory activity with IC_50_ values of 4.1 and 2.6 μmol/L, respectively,^6^ their inhibitory activities against other kinases have not been evaluated. Therefore, we evaluated the inhibitory activities of liphagal and siphonodictyal B against 313 kinases using a kinase panel assay. As shown in Figure S1 and Figure 1B, liphagal and siphonodictyal B had inhibitory activities against several kinases. In particular, liphagal and siphonodictyal B strongly inhibited activities against cyclin‐dependent kinase 4 (CDK4), CDK6, CDK7, and proto‐oncogene proviral integration site for murine leukemia virus‐2 (PIM‐2). The IC_50_ values of liphagal and siphonodictyal B against CDK4, CDK6, CDK7, and PIM2 are shown in Table 1.

**Table 1:** Evaluation of kinases inhibitory activities of liphagal or siphonodictyal B

Kinase	CDK4/Cyclin D3	CDK6/Cyclin D3	CDK7/Cyclin H/	PIM2	PIK3CA/PIK3R1^a^
			MAT1		
Liphagal	19.9	6.78	1.27	5.31	4.12
Siphonodictyal B	32.9	69.1	13.7	6.99	2.62

Kinase inhibitory activities were measured by the off‐chip mobility shift assay. IC_50_ (μmol/L) value was shown.

^a^ Kikuchi T, Narita K, Saijo K, Ishioka C, Katoh T. Enantioselective Total Synthesis of (−)‐Siphonodictyal B and (+)‐8‐epi‐Siphonodictyal B with Phosphatidylinositol 3‐Kinase *α* (PI3K*α*) Inhibitory Activity. 2016;2016(34):5659‐66.

### 3.2 Cytotoxic effects of liphagal and siphonodictyal B against human colon cancer cell lines

**Figure 2:**
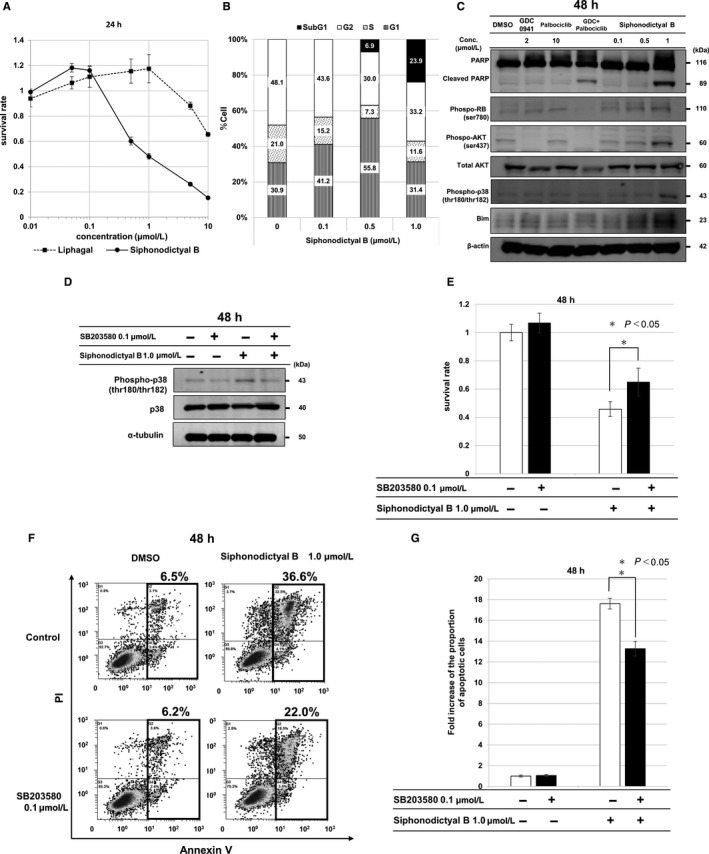
Effects by siphonodictyal B on cell proliferation, the cell cycle, and apoptosis. A, The antiproliferative effects of liphagal and siphonodictyal B. HCT 116 cells were treated with liphagal or siphonodictyal B at a concentration of 0.01, 0.05, 0.1, 0.5, 1, 5, or 10 μmol/L for 24 h. The ratios of surviving cells to control cells treated with 0.1% DMSO were calculated. Data are the means ± SD of three independent experiments performed triplicate. B, Flow cytometric analysis of the cell cycle. The G1, S, G2/M, and sub‐G1 phase populations are shown. HCT 116 cells were treated with siphonodictyal B at a concentration of 0.1, 0.5, or 1.0 μmol/L for 24 h. Siphonodictyal B induced G1 arrest and increased the sub‐G1 population. C, Western blot analysis of cells treated with 2 μmol/L GDC0941, 10 μmol/L palbociclib, a combination of 2 μmol/L GDC0941 and 10 μmol/L palbociclib (GDC + palbociclib), or siphonodictyal B (0.1, 0.5, or 1 μmol/L) for 48 h. Cleaved PARP, phospho‐AKT, phospho‐RB, phospho‐p38, and Bim were analyzed. D, Western blot analysis of phospho‐p38 expression in cells treated with siphonodictyal B with or without SB203580 for 48 h. E, Antiproliferative effects of siphonodictyal B with or without SB203580 for 48 h. The ratios of surviving cells to control cells were calculated. Data are the means ± SD of three independent experiments performed triplicate. *P < 0.05 compared with cells treated with siphonodictyal B alone. F, Flow cytometric analyses of apoptosis using annexin V and PI double staining in HCT 116 cells. HCT 116 cells were treated with siphonodictyal B with or without SB203580 for 48 h. The percentages of apoptotic cells are shown. G, The bar charts representing the proportion of apoptotic cells expressed as the fold increase vs that for untreated cells. Data are the means ± SD of three independent experiments. *P < 0.05 compared with cells treated with siphonodictyal B alone

We performed cell proliferation assays to evaluate the cytotoxic effects of liphagal and siphonodictyal B on human colon cancer cell lines. Siphonodictyal B exhibited more potent cytotoxic effects than liphagal in HCT 116 cells (Figure 2A). Similar results were also shown in HT‐29 and SW480 cells (Figure S2). Based on these results, we focused on siphonodictyal B in subsequent experiments.

### 3.3 The effects of siphonodictyal B on the cell cycle in HCT 116 cells

We performed FACS analyses to evaluate the effects of siphonodictyal B on the cell cycle. As shown in Figure 2B, both the G1 and sub‐G1 populations were slightly increased by treatment with 0.5 μmol/L siphonodictyal B. Moreover, the sub‐G1 phase was clearly increased by treatment with 1.0 μmol/L siphonodictyal B. These results revealed that siphonodictyal B induces apoptosis at higher concentrations in HCT 116 cells.

### 3.4 Siphonodictyal B induces apoptosis in HCT 116 via the induction of Bim

We performed Western blotting to investigate the molecular mechanism of apoptosis induced by siphonodictyal B. As shown in Figure 2C, treatment with 0.5 or 1.0 μmol/L of siphonodictyal B increased the levels of the apoptosis‐promoting protein Bim concentration dependently in HCT 116 cells. Treatment with 1.0 μmol/L siphonodictyal B also induced PARP cleavage. These results suggested that apoptosis induced by siphonodictyal B is attributable to the induction of Bim. Although siphonodictyal B has PI3K and CDK4/6 inhibitory activity, it did not inhibit AKT phosphorylation at Ser473 or RB phosphorylation at Ser780 in HCT 116 cells. By contrast, the PI3K inhibitor GDC0941 inhibited AKT phosphorylation. The CDK4 inhibitor palbociclib inhibited RB phosphorylation. Moreover, the combination of GDC0941 and palbociclib increased PARP cleavage. From these results, it was assumed that inhibitory activities against PI3K and CDK4/6 did not contribute to apoptosis induced by siphonodictyal B.

### 3.5 Siphonodictyal B induces apoptosis through p38 phosphorylation

It was previously reported that apoptosis is induced through activation of the p38 MAPK pathway in HCT 116 cells.^12^, ^13^ Therefore, we performed Western blotting to evaluate the effects of siphonodictyal B on the p38 MAPK pathway. As shown in Figure 2C, treatment with 1.0 μmol/L siphonodictyal B for 48 hour clearly induced p38 phosphorylation. Addition of the p38‐specific inhibitor SB203580 to siphonodictyal B resulted in reduced p38 phosphorylation (Figure 2D). In the cell proliferation assay, the addition of SB203580 partially canceled the cytotoxic effect of siphonodictyal B (Figure 2E). Likewise, in the annexin V‐PI double staining assay, the population of apoptotic cells induced by siphonodictyal B was attenuated by the addition of SB203580 (Figure 2F,G).

Moreover, the contribution of the p38 pathway was further validated via an RNAi approach. The protein level of phosphorylated p38 was significantly lower in cells treated with p38 siRNA (si#1 and si#2) than in cells treated with control RNA (siScr) (Figure S2A). Depletion of p38 partially reduced the cytotoxic effect of siphonodictyal B (Figure S2B). As expected, the annexin V‐PI double staining assay revealed that the induction of apoptosis by siphonodictyal B was lower in cells treated with p38 siRNA than in cells treated with control siRNA (Figure S2C,D). From these results, it was considered that in HCT 116 cells, siphonodictyal B induced apoptosis partially through the induction of p38 phosphorylation.

### 3.6 Siphonodictyal B induced apoptosis in HCT 116 cells by increasing ROS production

**Figure 3:**
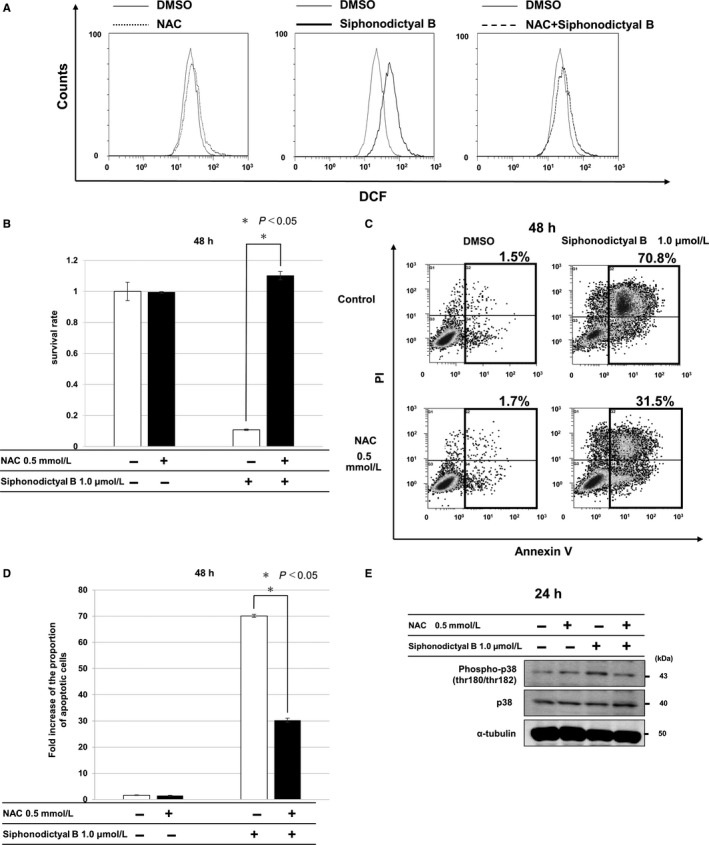
Elevation of intracellular ROS levels by siphonodictyal B. A, Flow cytometry of intracellular ROS levels using DCFDA staining in HCT 116 cells. HCT 116 cells were treated with siphonodictyal B with or without NAC for 2 h. B, Antiproliferative effects of siphonodictyal B with or without NAC for 24 h. The ratios of surviving cells to control cells were calculated. Data are the means ± SD of three independent experiments performed triplicate. *P < 0.05 compared with cells treated with siphonodictyal B alone. C, Flow cytometry of apoptosis using Annexin V and PI double staining in HCT 116 cells. HCT 116 cells were treated with siphonodictyal B with or without NAC for 24 h. The percentages of apoptotic cells are shown. D, The bar charts express the proportion of apoptotic cells as fold increases vs the number of cells treated with 0.1% DMSO. Data are the means ± SD of three independent experiments. *P < 0.05 compared with cells treated with siphonodictyal B alone. E, Western blotting of phospho‐p38 in cells treated with siphonodictyal B with or without NAC for 24 h

It had been reported that several terpenoids increased ROS levels and subsequently induced apoptosis in cancer cells.^14^, ^15^, ^16^, ^17^ We performed 2′,7′‐dichlorofluorescin diacetate (DCFDA) staining and FACS analysis to evaluate whether apoptosis induction by siphonodictyal B is related to ROS production. As shown in Figure 3A, ROS levels were higher in cells treated with siphonodictyal B than in those treated with DMSO. However, increased ROS production induced by siphonodictyal B was completely canceled by the addition of NAC, a scavenger of ROS (Figure 3A). The cytotoxic effects induced by siphonodictyal B alone were almost completely rescued by the addition of NAC (Figure 3B). Furthermore, the population of apoptotic cells induced by siphonodictyal B was greatly reduced by the addition of NAC (Figure 3C,3). These results indicated that siphonodictyal B induced apoptosis through the induction of ROS production in HCT 116 cells. The upregulation of phosphorylated p38 by siphonodictyal B was reduced by NAC (Figure 3E). These results suggested that siphonodictyal B induced apoptosis through the increase of ROS production and subsequent activation of the p38 MAPK pathway in HCT 116 cells.

### 3.7 Evaluation of the in vivo antitumor activity of siphonodictyal B

**Figure 4:**
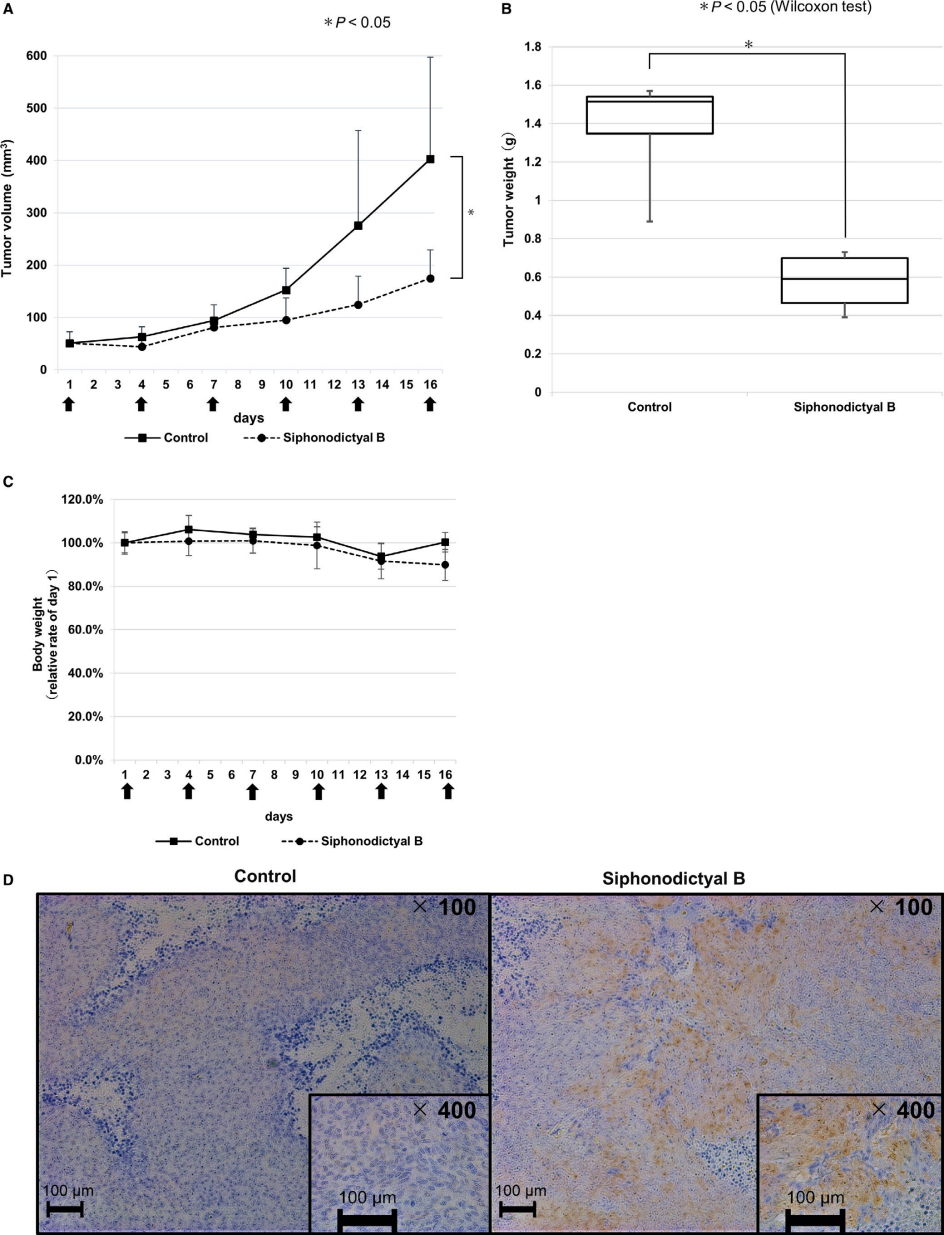
Antitumor activity of siphonodictyal B in vivo. (A‐C) HCT116 cells were implanted subcutaneously into the right flanks of mice. Mice were treated with siphonodictyal B (20 mg/kg) or vehicle via intraperitoneal injection every 3 d. Four mice were used for each treatment group. The tumor volumes in mice treated with vehicle or siphonodictyal B. The siphonodictyal B injection times are shown by black arrows. Data are the means ± SD from four mice. *P < 0.05 compared with the control group. Tumor weights were measured and represented by the boxplot. Data are the medians ± SD from four mice. *P < 0.05 compared with the control group. Body weights of mice were measured every 3 days. The relative ratios to the initial weights are shown. The siphonodictyal B injection times are shown by black arrows. Data are the means ± SD from four mice. Activation of the p38 MAPK pathway induced by siphonodictyal B in vivo. The in vivo biologic activity of siphonodictyal B was evaluated via immunohistochemistry using an antibody against phospho‐p38

Antitumor efficacy was evaluated using HCT 116 xenograft mouse models. Mice were divided in two groups (control group: solvent was intraperitoneally administered every 3 days and siphonodictyal B group: 20 mg/kg of siphonodictyal B was intraperitoneally administered every 3 days). As shown in Figure 4A,B, the tumor volume and weight were both significantly smaller in the siphonodictyal B group than in the control group. Body weights of mice in the siphonodictyal B group were similar to those in control mice (Figure 4C).

To confirm the induction of p38 MAPK pathway activation by siphonodictyal B in tumor tissue, tumors were isolated from mice after all treatments. The status of phosphorylated p38 in tumor tissue was immunohistochemically determined using the specific antibody. As shown in Figure 4D, tumor tissue in the siphonodictyal B group was strongly stained by the phosphorylated p38 antibody compared to the findings in the control group. This result verified the pharmacodynamic effects of siphonodictyal B in vivo, which is consistent with p38 MAPK pathway activation in vitro.

## 4 DISCUSSION

In the present study, we demonstrated that the terpenoid siphonodictyal B might induce apoptosis in colon cancer cells via ROS‐p38 MAPK pathway activation in vitro and inhibited tumor growth in vivo.

We observed that siphonodictyal B had kinase inhibitory activity against CDK4 and PI3K using the off‐chip mobility shift assay. However, siphonodictyal B did not reduce the expression levels of phosphorylated AKT and phosphorylated RB. It is known that mTORC1 and S6K regulate the negative feedback loop that attenuates insulin/IGF receptor signaling. mTORC1/S6K inhibitors suppress this negative feedback loop, thus upregulating the PI3K/AKT and ERK pathways.^18^ Similarly, as this example, cell signaling pathways complicatedly interfere with each other. We speculated that the inhibitory effects of siphonodictyal B on individual kinases were masked because the multitarget effects of the compound might influence multiple signaling pathways that interfere with each other, although we did not pursue this issue in this study.

Both liphagal and siphonodictyal B induced cell growth inhibitory activity in a concentration‐dependent manner. Although it was already known that liphagal inhibited cell proliferation,^4^ the effect of siphonodictyal B on cell proliferation had not been previously studied as far as we know. We elucidated that siphonodictyal B exhibits more potent cell growth inhibitory activity than liphagal.

5‐FU, irinotecan, and oxaliplatin, cetuximab (antiepidermal growth factor receptor antibody), and aflibercept (vascular endothelial growth factor antagonist) are key drugs for colon cancer chemotherapy.^19^, ^20^, ^21^, ^22^ Previous studies reported that mutation of *RAS, BRAF, TP53*, and *PIK3CA* is involved in resistance to these drugs.^23^, ^24^, ^25^, ^26^ HCT 116 (*KRAS* G13D mutant*, BRAF* wild type*, TP53* wild type, and *PIK3CA* H1047R mutant), HT‐29 (*KRAS* wild type, *BRAF* V600E mutant, *TP53* R237H mutant, and *PIK3CA* P499T mutant), and SW480 (*KRAS* G12V mutant, *BRAF* wild type, *TP53* R237H mutant, and *PIK3CA* wild type) cells were used to evaluate the anticell proliferation activity of siphonodictyal B, which was similar in the three cell lines (Figure S2).These results suggest that anticell proliferation effect of siphonodictyal B is independent of these genes, it is expected that siphonodictyal B might overcome these resistances.

In normal cells, apoptosis is strictly regulated to maintain a healthy balance of survival and death.^27^ Apoptosis occurs via activation of the caspase family through intrinsic and extrinsic apoptotic pathways. The intrinsic pathway controls caspase family activation through the expression of the proapoptotic (eg, Bim, BAK, BAX) and antiapoptotic proteins (eg, BCL‐2, MCL‐1, XIAP).^28^ In this study, we found that siphonodictyal B induced apoptosis via upregulation of Bim.

Terpenoids such as echnocystic acid, dehydrocostus lactone, and furanodiene have the ability to induce apoptosis through p38 MAPK pathway activation.^29^ We found that downregulation of the p38 MAPK pathway at least partially attenuated siphonodictyal B‐induced apoptosis in HCT 116 cells. The result showed that siphonodictyal B induces apoptosis at least in part through p38 MAPK pathway activation.

Previous research found that the p38 MAPK pathway was activated by various extracellular or endogenous cellular stimuli. ROS represent a major trigger of the p38 MAPK pathway.^30^ Moreover, it is known that moderate levels of ROS play an important role in healthy cell proliferation and differentiation.^31^ However, excessive ROS levels reaching the toxic threshold result in irreversible damage to intracellular molecules, such as lipids, proteins, and nucleic acids.^32^ Therefore, the antioxidant defense system tightly controls ROS generation in normal cells. Conversely, ROS production is upregulated in cancer cells, and elevated ROS levels are associated with abnormal cell proliferation. However, excessive levels of ROS also damage cancer cells. Therefore, if there is a drug that modulates a balance of ROS generation and elimination, it may be a novel anticancer drug with a few side effects that is less toxic to normal cells.^33^ In the present study, siphonodictyal B markedly promoted ROS generation and induced apoptosis.

As shown in Figure 4B,E, NAC almost completely reversed both cell growth inhibition and p38 phosphorylation induced by siphonodictyal B. However, when the p38 MAPK pathway was downregulated via p38 inhibition or depletion, the cytotoxic and apoptosis‐inducing effects of siphonodictyal B were partially attenuated. These results also suggest that the p38 MAPK pathway is involved downstream of ROS signaling as part of the mechanism of siphonodictyal B‐induced apoptosis in HCT 116 cells.

Also, siphonodictyal B significantly suppressed tumor growth in vivo. We preliminarily investigated the antitumor effects of siphonodictyal Bintraperitoneally administrated at a dosage of 20 mg/kg. The antitumor effect is expected to be further enhanced by optimizing the administration route, frequency, and dosage in the future. We hope that siphonodictyal B might become a promising seed of novel anticancer drug.

## CONFLICT OF INTEREST

The authors have no conflicts to disclose in relation to this work.

## DATA AVAILABILITY STATEMENT

The data that support the findings of this study are available from the corresponding author upon reasonable request.
