# Supplementary figures and images for: In Vitro and in Vivo antitumor activity and the mechanism of siphonodictyal B in human colon cancer cells
Source: Cancer Med. 2019 Jul 31;8(12):5662–72. doi: 10.1002/cam4.2409 (PMC6745845; doi:10.1002/cam4.2409)

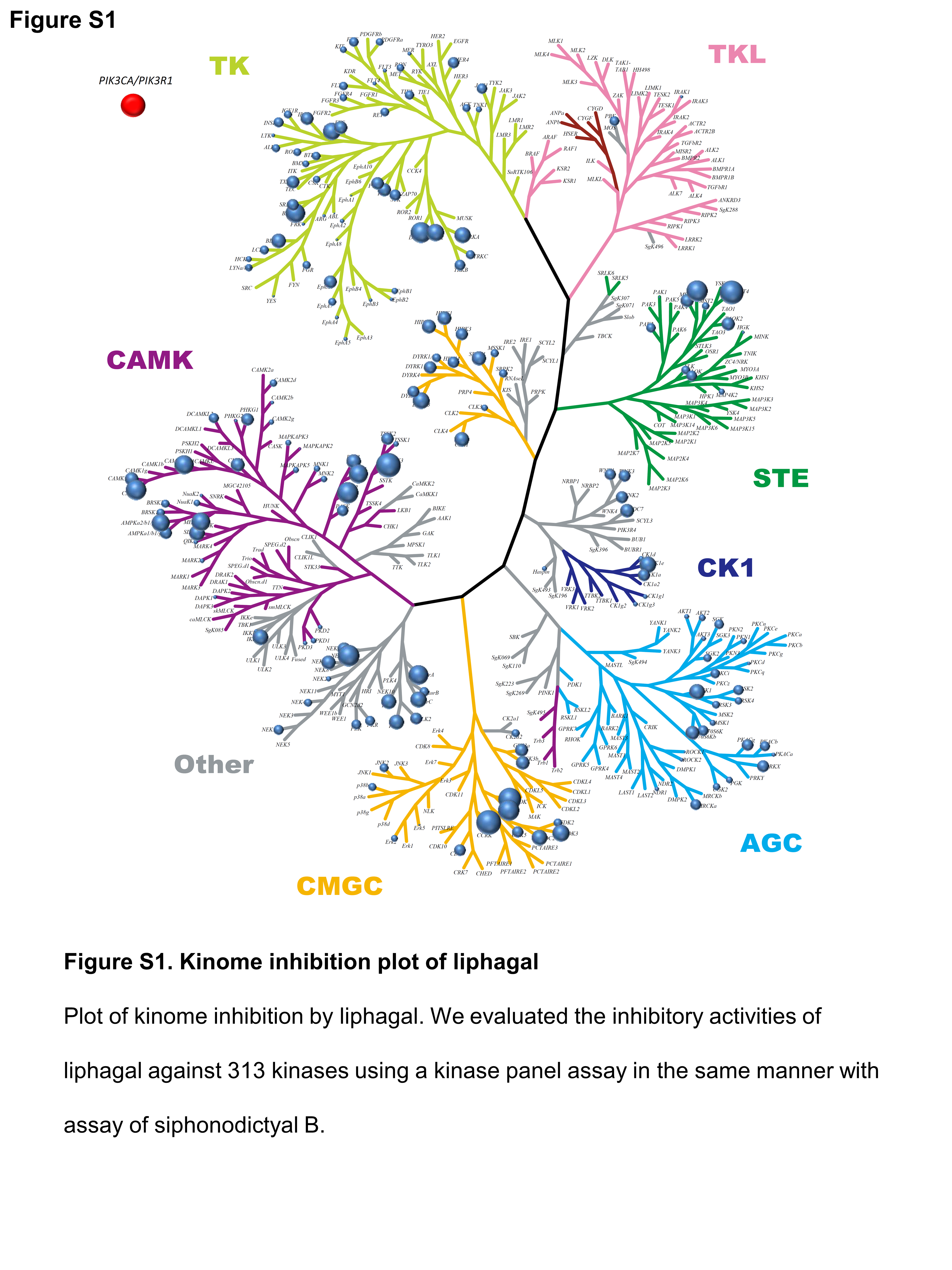

Supplement: Supplementary file 1 [file CAM4-8-5662-s001.TIF]

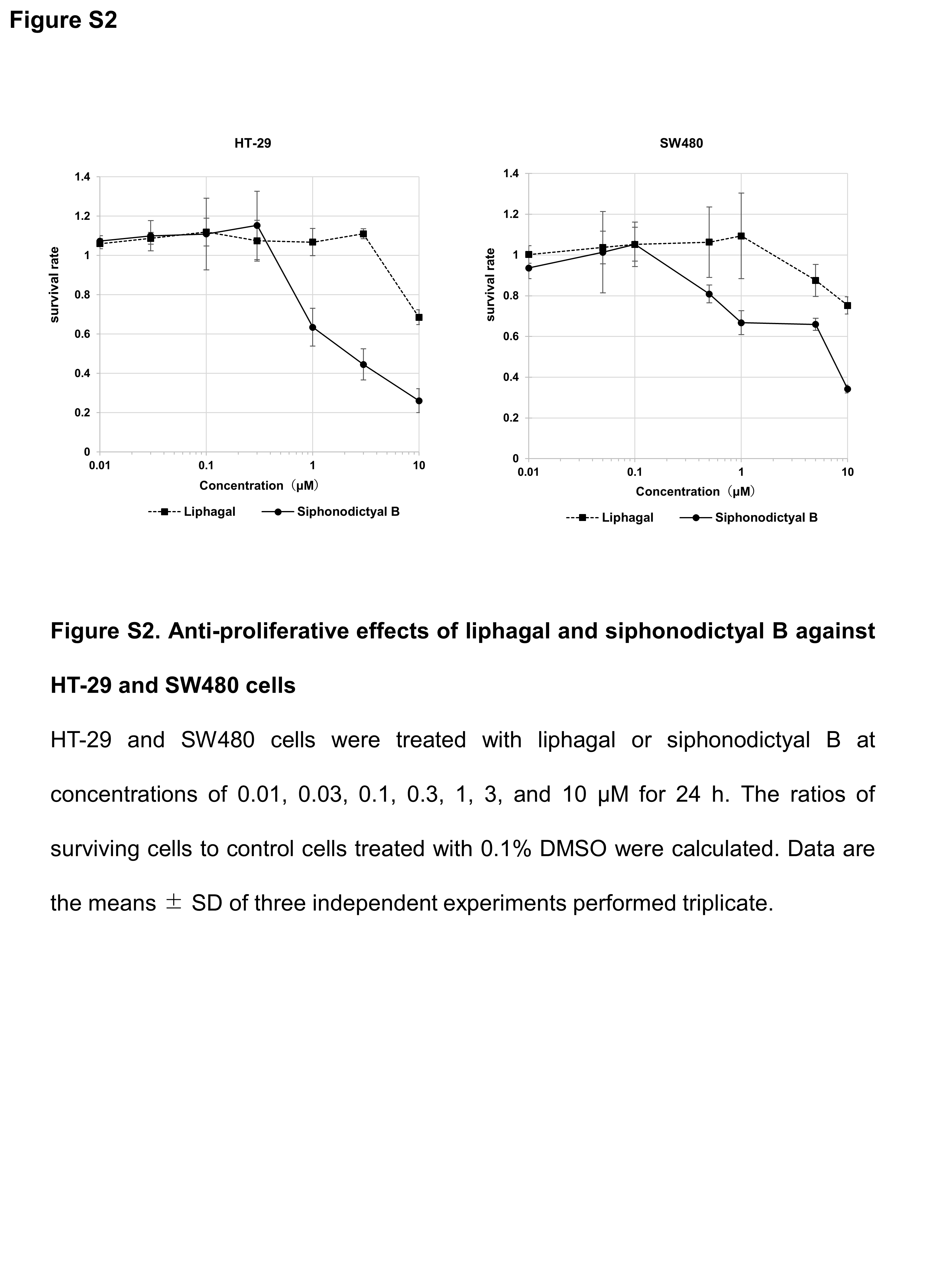

Supplement: Supplementary file 2 [file CAM4-8-5662-s002.TIF]

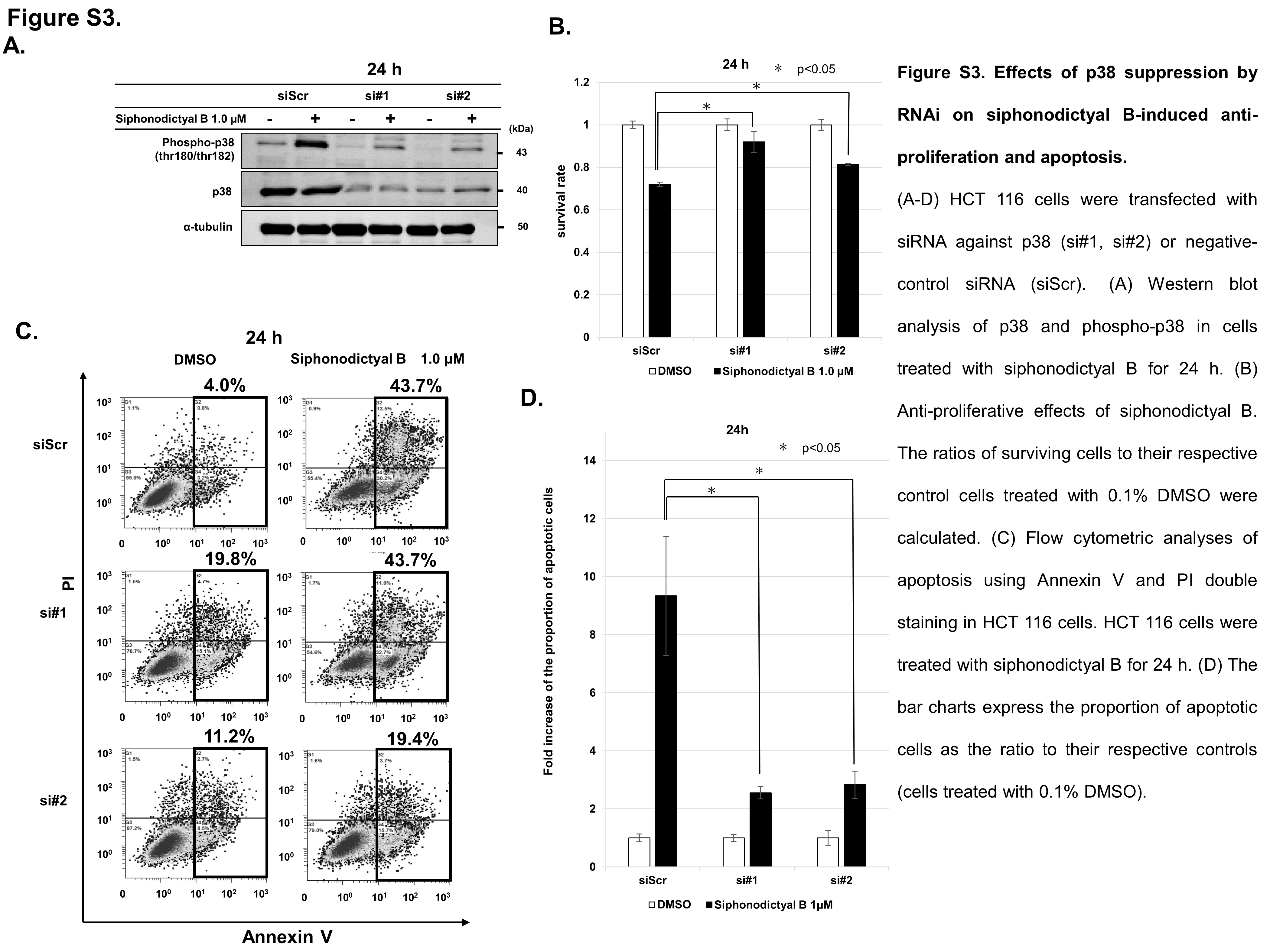

Supplement: Supplementary file 3 [file CAM4-8-5662-s003.tif]

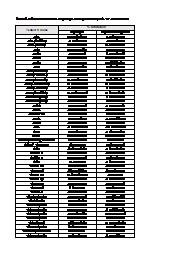

Supplement: Supplementary file 4 [file CAM4-8-5662-s004.docx › docProps/thumbnail.jpeg]
